# Collapse of Telomere Homeostasis in Hematopoietic Cells Caused by Heterozygous Mutations in Telomerase Genes

**DOI:** 10.1371/journal.pgen.1002696

**Published:** 2012-05-17

**Authors:** Geraldine Aubert, Gabriela M. Baerlocher, Irma Vulto, Steven S. Poon, Peter M. Lansdorp

**Affiliations:** 1 Terry Fox Laboratory, British Columbia Cancer Agency, Vancouver, British Columbia, Canada; 2 Experimental Hematology, Department of Clinical Research, University of Bern, Bern, Switzerland; 3 Division of Hematology, Department of Medicine, University of British Columbia, Vancouver, British Columbia, Canada; 4 European Research Institute for the Biology of Ageing, University of Groningen, University Medical Centre Groningen, Groningen, The Netherlands; Stanford University School of Medicine, United States of America

## Abstract

Telomerase activity is readily detectable in extracts from human hematopoietic stem and progenitor cells, but appears unable to maintain telomere length with proliferation *in vitro* and with age *in vivo*. We performed a detailed study of the telomere length by flow FISH analysis in leukocytes from 835 healthy individuals and 60 individuals with reduced telomerase activity. Healthy individuals showed a broad range in average telomere length in granulocytes and lymphocytes at any given age. The average telomere length declined with age at a rate that differed between age-specific breakpoints and between cell types. Gender differences between leukocyte telomere lengths were observed for all cell subsets studied; interestingly, this trend could already be detected at birth. Heterozygous carriers for mutations in either the telomerase reverse transcriptase (*hTERT*) or the telomerase RNA template (*hTERC*) gene displayed striking and comparable telomere length deficits. Further, non-carrier relatives of such heterozygous individuals had somewhat shorter leukocyte telomere lengths than expected; this difference was most profound for granulocytes. Failure to maintain telomere homeostasis as a result of partial telomerase deficiency is thought to trigger cell senescence or cell death, eventually causing tissue failure syndromes. Our data are consistent with these statements and suggest that the likelihood of similar processes occurring in normal individuals increases with age. Our work highlights the essential role of telomerase in the hematopoietic system and supports the notion that telomerase levels in hematopoietic cells, while limiting and unable to prevent overall telomere shortening, are nevertheless crucial to maintain telomere homeostasis with age.

## Author Summary

Human blood cells all originate from a common precursor, the hematopoietic stem cell. Telomerase, the enzyme responsible for adding telomere repeats to chromosome ends, is active in human hematopoietic stem cells but appears unable to maintain a constant telomere length with age. We first document the telomere length of different blood cell subsets from 835 healthy individuals between birth and 100 years, to delineate the normal rate of telomere attrition with age. Telomere lengths of blood cells were found to be slightly longer in women than in men, from birth and throughout life. We then compared this reference data to the telomere length in similar blood cell subsets from individuals with reduced telomerase activity as a result of a mutation in one of the genes encoding telomerase and from their direct relatives. Strikingly short telomeres were found in telomerase-deficient individuals, consistent with their cellular pathology and disease susceptibility, and somewhat shorter telomeres than expected were found in cells of relatives with normal telomerase maintenance. Our data can be used as a reference for blood cell telomere length in studies of normal and accelerated aging.

## Introduction

At least a few hundred nucleotides of telomere repeats must “cap” each chromosome end in order to suppress DNA damage signals and avoid the activation of DNA repair pathways [1]–[3]. Critically short or “uncapped” telomeres may be repaired by the enzyme telomerase [4] or by recombination [5]. However, the capacity of these telomere repair processes appears limited in most human somatic cells [6]. Apoptosis or cellular senescence is triggered when too many “uncapped” telomeres accumulate [7], posing a barrier to tumor growth, but also contributing to loss of cells with age [8].

Despite increasing evidence that telomere homeostasis is important in human aging, cancer and disease states, detailed and comparative information regarding the telomere length in different human cell subtypes of healthy individuals in relation to their age is surprisingly modest. Apart from being technically challenging [9] such studies are complicated because at birth and throughout life, telomere length is highly variable between chromosomes [10], [11], between cells [12], [13] and between individuals. Studies of identical twins have shown that individual differences in average telomere length appear to be largely genetically determined [14], [15].

In most somatic cells the telomere length declines with age and with cell division in culture, albeit at different rates [13], [16]. For example, in humans and baboons, lymphocytes show a more pronounced telomere loss with age than granulocytes [14], [17]. These two cell types represent the two major branches of the hematopoietic system, which can be further subdivided into distinct cell populations based on their phenotype and function. Within the hematopoietic hierarchy, the most primitive cells, hematopoietic stem cells (HSC), have the longest telomeres [18], [19]. HSC differentiate to produce progenitor cells of both the myeloid and lymphoid lineage that proliferate prior to differentiation into mature “end” cells. Unlike most immune cells most differentiated myeloid cells such as granulocytes are incapable of further cell divisions.

The precise role of telomerase in hematopoietic stem and progenitor cells and in lymphocytes remains poorly understood. Telomerase expression is readily detected in hematopoietic cells [20]–[22]; however, this activity appears unable to prevent telomere loss with age or proliferation. It is often assumed that telomerase is required to maintain the telomere length in various stem cells. With the exception of embryonic stem cells and abnormal tumor (stem) cells this assumption is not supported by data. Studies on the role of telomeres and telomerase in HSC from healthy individuals are challenging because HSC are very rare cells that typically reside in bone marrow. In contrast, the various nucleated blood cells that are derived from HSC are easily accessible for study. The average telomere length in granulocytes can be used as a surrogate marker for the telomere length in HSC [23], if one assumes that the number of cell divisions between HSC and granulocytes is relatively constant [18]. Individual carriers of heterozygous mutations for either the telomerase RNA gene (*hTERC*) or the telomerase reverse transcriptase gene (*hTERT*) can present with a wide spectrum of diseases [24] including dyskeratosis congenita [25], [26], bone marrow failure [24] and pulmonary fibrosis [27]. Heritable telomerase deficiencies provide an excellent model to study the role of telomerase in human hematopoietic cells.

Here we report our data on the median telomere length (MTL) in five distinct leukocyte subpopulations of over 800 healthy individuals between birth and 100 years of age as well as 60 individuals that are heterozygous for one of the telomerase genes, *hTERC* or *hTERT*. The telomere length in leukocytes from healthy individuals was found to vary over a broad range at any given age and the rate of telomere attrition also varied with age and with cell type. Strikingly, the telomeres in cells from individuals with telomerase deficiency were found to be very short in all cell types, and this deficit was found to be comparable for most cell subtypes for *hTERC* or *hTERT* deficiency. The largest (age adjusted) differences in telomere length deficits between *hTERC* or *hTERT* were seen in “naïve” T cells for *hTERC* deficient individuals and in NK/differentiated T cells for *hTERT* deficient individuals. These results demonstrate that normal telomerase levels are essential to maintain normal telomere homeostasis in HSC and lymphocytes. Our results provide valuable reference data for further studies of telomere biology in health and disease and point to a crucial rate-limiting role for telomerase in HSC and immune cells.

## Results

### Lymphocyte and granulocyte telomere length dynamics with age

**Figure 1:**
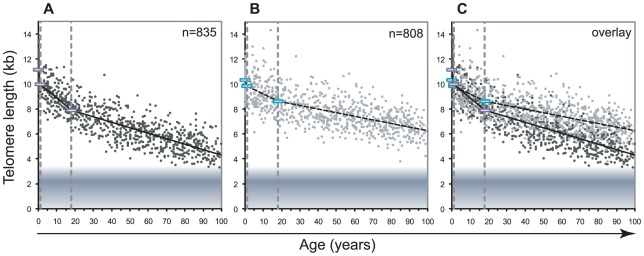
Decline in telomere length with age differs between lymphocytes and granulocytes. The median telomere length in nucleated blood cells from 835 healthy individuals ranging from birth (umbilical cord blood) to 102 years of age were measured by flow FISH. The results were used to calculate the telomere attrition over time using linear regression in three age segments. A. Median telomere length in lymphocytes (black dots). B. Median telomere length in granulocytes (grey dots). Breakpoints in the piece-wise linear regression lines are marked by rectangles and the three age groups are marked by dotted vertical grey lines at 1 and 18 years. On average 8 individuals were tested per age-year. C. At any given age, a wide range of telomere length was observed and the decline in telomere length with age in lymphocytes was more pronounced than in granulocytes. The shaded area represents the estimated length of subtelomeric DNA. Note that in older individuals, on average only 1–2 kb of telomere repeats were present in lymphocytes.

We measured the telomere length in lymphocytes and granulocytes of 835 healthy individuals using automated multicolor flow FISH (Figure 1). On average, 7 to 8 individuals were tested for each age-year. Various best-fit models were tested to model the overall decline in telomere length with age. In view of the very rapid decline in telomere length in the first years of life in humans [14] as well as non-human primates [28], we divided the telomere length decline over three age segments. The first is between birth and one year of age when the growth rate of bones and weight in infants shows a marked deceleration (for reference curves, see http://www.cdc.gov/growthcharts/clinical_charts.htm). A second arbitrary cut-off was set at 18 years of age because the decline in telomere length in all leukocytes appeared to drop notably after puberty. Telomere length data within the three selected age segments: below 1 year (yr), 1–18 yrs and 19 yr and higher are shown in Figure 1 and Table 1. The overall age-related telomere length decline was most pronounced in lymphocytes with significant losses ranging from 1190 base pairs (bp) per year between birth and 1 year of age to 126 bp per year during childhood and 43 bp per year in adulthood. In contrast, the age-related telomere length decline in granulocytes and by extension in HSC was more modest during early life (485 bp per year), childhood (74 bp per year) and adulthood (28 bp per year).

**Table 1:** Telomere length distribution and age-related telomere length decline in healthy individuals.

		lymphocytes	granulocytes	CD20+	CD45RA+ CD20−	CD45RA−	CD57+
**Slope (bp/yr) age range:**	**<1**	−1190	−485	−916	−947	−2073	−2818
	**1–18**	−126	−74	−70	−89	−144	−155
	**19–102**	−43	−28	−27	−51	−32	−39
**MTL regression (kb) age:**	**birth**	11.2	10.3	10.9	11.1	11.4	12.4
	**1**	10.0	9.9	9.9	10.1	9.3	9.6
	**18**	7.9	8.6	8.8	8.6	6.8	7.0
	**102**	4.3	6.2	6.5	4.3	4.1	3.7
**MTL range (kb)**	**1–99 percent**	4.34	4.83	4.67	5.02	4.09	5.19
	**10–90 percent**	2.45	2.67	2.37	2.80	2.28	2.89

**Figure 2:**
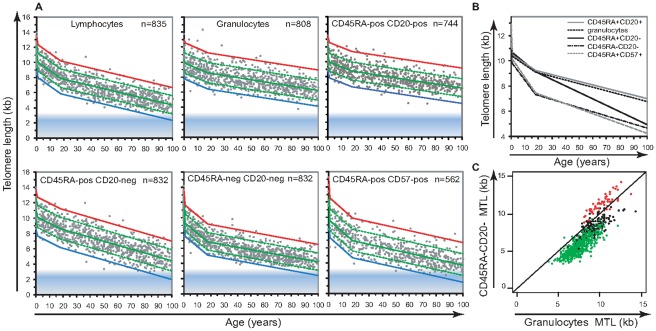
Cell type–specific differences in the range and attrition rate of leukocyte telomere length. A. Cross-sectional median telomere length in nucleated cell types determined by flow FISH in 835 healthy individuals over the age range from birth to 102 years of age. The following nucleated blood cell subtypes were analyzed : lymphocytes, granulocytes, CD45RA positive CD20 positive B lymphocytes (CD20+), CD45RA positive CD20 negative lymphocytes (CD45RA+pos CD20−) “naïve” T cells, CD45RA negative CD20 negative lymphocytes (CD45RA−) memory T cells and CD45RA positive CD57 positive mature NK/T cells (CD57+). Data were analyzed using a piece-wise linear regression model in the age categories 0, 1 yr; 18 yrs and 102 years and for calculation and representation of the telomere length distribution range at any given age (expressed as a percentile): 99^th^ (red), 90^th^ (dashed green top), 50^th^ (green), 10^th^ (dashed green bottom) and 1^st^ (blue). Some of the healthy subjects (n = 835) did not have sufficient cells for analysis of one or more of the cell subsets. B. Piece-wise linear regression analysis overlay representing the modeled estimate of telomere length per age for: B lymphocytes (grey), granulocytes (black dashed), “naïve” T lymphocytes (black), memory T lymphocytes (black interrupted dashed) and mature NK/T lymphocytes (grey dashed). Regression breakpoints were set at age 1 and age 18 years. C. Paired comparison of telomere length in granulocytes and memory T lymphocytes from the same individuals. Age groups were as follows: cord blood samples and below age 1 (n = 60, red), age 1 to 18 (n = 171, black), 19 and above 19 (n = 604, green).

Telomere length loss between the three selected age segments, results of the piece wise linear regression analysis for each age as depicted in Figure 1, Figure 2, and Figure 4 as well as telomere length ranges are summarized for each leukocyte subsets.

MTL median telomere length.

### The decline in telomere length with age varies between leukocyte subpopulations

Telomere length measurements versus age in granulocytes and lymphocyte subpopulations were used to determine the regression lines for telomere attrition in the three selected age ranges (regression estimates shown in Figure 2A; the complete data set can be accessed in Table S1). These regression lines were shifted according to data distribution (from the overall regression estimate) to represent the 99^th^, 90^th^, 10^th^ and 1^st^ percentile of the telomere length distribution in each age segment for each blood cell subset in healthy individuals. The rate of telomere length decline varied amongst the different lymphocyte subsets analyzed. The telomere length decline with age in B lymphocyte subset (CD45RA+ CD20+) was comparable to that in granulocytes. Memory T (CD45RA−CD20−) and mature NK/T (CD45RA+CD57+) lymphocyte subsets showed the sharpest decline in telomere length with age, particularly during childhood with slopes of −144 and −155 bp per year respectively. The CD45RA+CD20− T lymphocyte subset enriched for “naïve” T cells and the CD45RA+CD57+ mature NK/T lymphocyte subset displayed the widest distributions, 2.80 and 2.89 kilobase (kb) respectively between the 10^th^ and 90^th^ percentile of the normal distribution, throughout the age ranges. Unlike other subsets CD45RA+CD20− T lymphocytes showed only a modest difference in the telomere attrition rate between childhood and adulthood: 89 and 51 bp per year respectively. In contrast, the memory T lymphocyte subset (CD45RA−CD20−) displayed the narrowest range of telomere length distribution (2.28 kb between the 10^th^ and 90^th^ percentile of the normal distribution). Overall, the shortest telomere lengths were measured in memory T and mature NK/T lymphocytes from older individuals.

### Comparisons of telomere length in different leukocyte subsets

From our cross-sectional data, we determined the average telomere length decline with age for the different leukocyte subpopulations (Figure 2B). During childhood, granulocytes, CD45RA+CD20− “naïve” T lymphocytes and CD20+ B lymphocytes all showed a very similar decline in telomere length, whereas the rate of decline in memory T cells was much higher. Paired MTL values in different blood cell subsets from the same individual revealed that around one year of age the telomere length values in memory T lymphocytes drop below those of granulocytes (Figure 2C). In contrast, telomere length values in B lymphocytes remained comparable to those in granulocytes over the entire age range. One caveat in our measurement of telomere length in “naïve” (CD45RA+CD20−) T lymphocytes is that terminally differentiated effector lymphocytes re-expressing CD45RA are likely to represent an increased proportion within this cell population in older individuals. As a consequence, measurements within the subset of “naïve” T cells are variably skewed in older individuals (as illustrated by the direct comparison of MTL between “naïve” T lymphocytes and other cell subtypes from the same individual over 4 distinct age groups in Figure S2A and Table S2).

### Gender differences in leukocyte telomere length measurements

**Figure 3:**
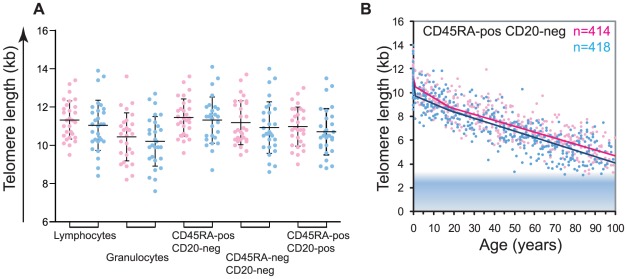
Gender differences in leukocyte telomere length. A. Telomere length measurements in leukocyte subsets from female (pink) versus male (blue) healthy newborns (n = 58; females n = 29, males n = 29). The following white blood cell subsets were tested lymphocytes, granulocytes, “naïve” T lymphocytes (CD45RA+CD20−), memory T lymphocytes (CD45RA−CD20−) and B lymphocytes (CD20+). Each dot represents an individual sample, mean (horizontal bar) and standard deviation (vertical bar) for each cell type are shown. All subsets display a trend for longer average telomere lengths in females; however this trend does not reach statistical significance (Student's t test, data not shown). B. Piece-wise linear regression analysis representing the calculated telomere length per age for females (pink) versus males (blue) “naïve” T lymphocytes (CD45RA+CD20−). Breakpoints were at 1 year of age and at 18 years. Analysis of variance for females versus males (statistical model in R) showed significance (F(4,825) = 9.05; P = 3.7×10^−7^, ANOVA test result comparing regression fits). For data on other subsets and details of statistical analysis, see Figure S3 and Table S5.

Measurements from cord blood samples provided the earliest opportunity to assess the telomere lengths in cells from healthy individuals. Interestingly, of all the cell types measured from cord blood, granulocytes showed the shortest telomeres at birth: differences were significant for granulocytes versus CD45−CD20− memory T lymphocytes and CD45+CD20− “naïve” T lymphocytes but not for granulocytes versus B lymphocytes. Comparisons were tested by one-way ANOVA (n = 58): F(5,265) = 5.7; P = 0.0002, Table S3, followed by Tukey's multiple comparison test, see details in Table S4). Interestingly, female newborns appeared to have longer telomeres than males (Figure 3A); however this trend did not reach statistical significance. Further comparisons of telomere length in leukocyte subsets as a function of gender showed highly significant differences between males and females in the CD45RA+CD20− “naïve” T lymphocyte subset over the entire age range (F(4,825) = 9.05; P = 3.7×10^−7^, ANOVA test result comparing regression fits; Figure 3B and Table S5). Significant differences were also seen for other leukocyte subsets in each age segment with the exception of granulocytes and memory T lymphocytes, which displayed similar, average telomere lengths after 18 years of age (Figure S3 and Table S5).

### Telomerase is essential to maintain leukocyte telomere length homeostasis

**Figure 4:**
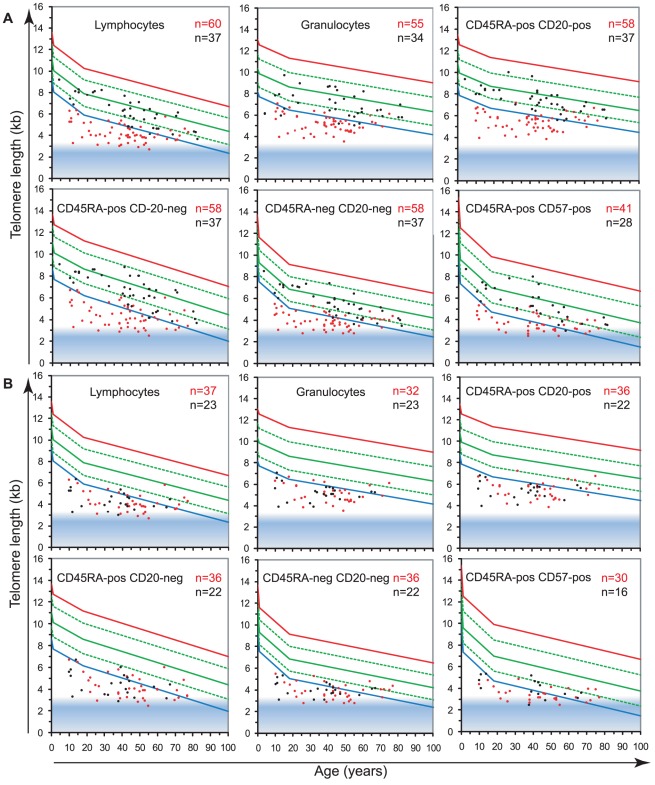
Telomerase is essential to maintain telomere length in leukocytes. The telomere length distribution in healthy individuals (Figure 2) was used to plot the median telomere length values of leukocyte subsets obtained with: A. leukocytes from 60 telomerase deficient patients (red) and 37 non-carrier relatives (black). B. leukocytes from 37 *hTERT* mutation heterozygous individuals (red) and 23 *hTERC* mutation heterozygous carriers (black).

To study the role of telomerase in hematopoietic cells, we analyzed the telomere length in leukocyte subpopulations of individuals carrying a mutation in either *hTERT* or *hTERC* (n = 60) in comparison to non-carrier relatives (n = 37). The results, plotted on the telomere length versus age distribution curves derived from healthy individuals (Figure 2A) are shown in Figure 4 and Table 1. Strikingly, telomerase heterozygous individuals showed very short telomeres (typically below the 1^st^ percentile of the normal distribution) at all ages and for all blood cell subsets tested (ANOVA test P<2.2×10^−16^, for full details of analyses see Table S5). The shortest telomeres were measured in mature NK/T cells (mean of 3.7±0.7 kb for all telomerase heterozygous individuals not adjusted for age, Figure 4A) and the CD45RA+CD20− “naive” lymphocyte subset appeared the most severely impacted by telomerase deficiency with an average difference to the normal distribution (adjusted for age) of Δtel: 3.2 kb. Differential analysis of leukocyte telomere lengths in leukocytes from *hTERT* vs. *hTERC* heterozygous individuals showed a similar effect on most blood cell subtypes (Figure 4B and Table 2). Exceptions were an increased telomere loss in the CD45RA+CD57+ mature NK/T cells (difference of 0.4 Kb) of *hTERT* deficient individuals (n = 37) and a slightly increased effect (difference of 0.2 Kb) on the CD45RA+CD20− “naive” lymphocyte subset for *hTERC* deficient individuals (n = 23).

**Table 2:** Telomere loss corrected for age (Δtel) in telomerase heterozygous individuals and unaffected relatives.

Δtel (kb)§	lymphocytes	granulocytes	CD20+	CD45RA+ CD20−	CD45RA−	CD57+
***Tel*** ** +/− individuals (60)**	−2.7	−2.9	−2.8	−3.2	−2.3	−2.4
***TERT +/−*** ** individuals (37)**	−2.6	−2.9	−2.7	−3.1	−2.3	−2.5
***TERC*** ** +/− individuals (23)**	−2.8	−2.9	−2.9	−3.3	−2.3	−2.1
***Tel*** ** +/+ relatives (36)**	−0.6	−0.9	−0.6	−0.7	−0.6	−0.6
***Tel +/+*** ** parent (7)**	−0.9	−0.8	−0.8	−1.1	−0.8	−0.6
***Tel +/+*** ** sibling (8)**	−0.7	−0.7	−0.8	−0.9	−0.8	−0.6

Summary of telomere loss adjusted for age (Δtel) in telomerase heterozygous individuals considered as a group (*Tel*), considered as *TERT* or *TERC* heterozygous individuals separately, and unaffected relatives of telomerase heterozygous individuals considered as a group (*Tel*), or separating parents or siblings.

§ Difference between the median telomere length and the regression estimate of the telomere length distribution in healthy individuals of the same age (Δtel).

*Tel*: *hTERC* or *hTERT* gene.

Non-carrier relatives of telomerase deficient individuals (*hTERT* and *hTERC* considered together), despite having intact telomerase genes, also showed somewhat shorter median telomere lengths in all leukocytes compared to the control population. The largest difference was measured in the granulocyte subset of non-carrier relatives considered together, with Δtel: 0.9 kb, which may be indicative of HSC deficit and warrants further investigation (Figure 4A; ANOVA test: F(3,837) = 8.1; P = 2.63×10^−5^, for full details of analyses see Table S5). Both parents and siblings of heterozygous individuals were found to have slightly shorter telomere lengths for age (Table 2 and Figure S4). Differential analysis of parents (n = 6) and siblings (n = 4) of *hTERT* deficient individuals was also performed and showed comparable telomere length deficits for all cell subsets tested (Figure S4 and data not shown) in this relatively small group.

## Discussion

In this report, we show telomere length data for five distinct leukocyte subpopulations from over 800 healthy individuals, representing a comprehensive and representative cross-sectional analysis of telomere length in leukocyte subpopulations over the entire human life span. The value of this data is illustrated by our analysis of individuals with heritable telomerase deficiencies. Leukocyte telomere length was found to clearly distinguish between relatives with and without mutations in *hTERT or hTERC* supporting telomere length measurements as a screen for mutations in “telomere maintenance” genes. Our results confirm and extend earlier reports of telomere loss in leukocytes with age [13], [14] and document a crucial role for telomerase in controlling leukocyte telomere length.

Telomerase expression is readily detected in most hematopoietic cells [20]–[22], yet this activity appears unable to prevent the overall loss of telomeric DNA with age or proliferation. Most likely, telomerase is primarily required to directly act on chromosome ends in hematopoietic cells themselves, however secondary, indirect effects of telomerase via cells that support cell proliferation [29] or possible effects of the TERT protein on transcription in stem cells [30] are difficult to exclude.

Heterozygosity for one of the telomerase genes, expected to reduce telomerase levels by half, results in a striking telomere deficit (Figure 4). How can this finding be explained? One possibility is that the primary function of telomerase in somatic cells is the repair [8] or protection [31] of critically short telomeres. Failure to properly “cap” all chromosome ends with telomere repeats results in activation of a DNA damage response [1], [32]. Detrimental consequences for HSCs and lymphocytes could result when DNA damage signals from uncapped telomeres persist or reach a certain threshold and cause apoptosis of such cells. Impaired “capping” of telomeres in cells with reduced telomerase could affect telomere length directly and indirectly. Direct effects on telomere length could result from normal replication of telomeric DNA [8] and damage caused by reactive oxygen species [33]–[35]. Indirect effect on telomere length would result from the additional cell division required to compensate for the increased cell losses. Compensatory cell divisions in cells from telomerase deficient individuals could be particularly taxing as more short telomeres are expected to emerge with each extra cell division. The resulting feed-forward loop could exhaust the stem cell compartment in infants and children explaining the marrow failure typically seen in pediatric telomerase deficient patients. In cases where sufficient stem cells survived till adulthood, the same unproductive feed-forward loop could exhaust cells of the immune system. This possibility is in line with the observation that after puberty telomere attrition in more mature subsets of T and NK cells is notably higher than in granulocytes as a surrogate marker for stem cells (Figure 2). We speculate that the balance between end cells such as granulocytes and macrophages on the one hand and various other immune cell types on the other is perturbed in older telomerase deficient patients. Such an imbalance could result in failure to clear pathogens and immunogens and create pro-fibrotic conditions or result in failure to remove senescent cells [36].

Apart from cell turnover and telomerase levels, the telomere length in parental chromosomes at fertilization is another probable variable in the disease manifestations of telomerase deficient patients. This variable will determine when critically short telomeres, requiring repair or capping by telomerase, will appear: during development or during adult life. This notion is in line with the age-related onset of symptoms or “anticipation” in multi-generation telomerase deficiency disorders [24], [37] and our observation that telomeres in cells from unaffected children and parents of telomerase heterozygous individuals are somewhat shorter than expected (Figure 4 and Table 2).

As was shown previously for human lymphocytes and granulocytes [14], [38] and confirmed in longitudinal studies of non-human primates [28], leukocyte telomere length shortens most dramatically very early in life. This rapid decline can be explained by steady proliferation of stem cells and immune cells after birth. After one year of age we observed a rapid deceleration in telomere loss most likely reflecting an intrinsic, ontogeny-related change in stem cell turnover and function [39], which has also been observed in postnatal mice [40], [41]. Our observations with human and primate cells suggest that each HSC cell division in these species is “counted” by the loss of telomeric DNA. Why a relative modest decline in telomerase activity in humans results in a wide spectrum of diseases whereas complete loss of telomerase is typically tolerated for several generations in yeast, plants, worms and mice remains incompletely understood [42].

The shortest overall telomere lengths were measured in the mature NK/T cell subsets of older healthy individuals and of *hTERT* telomerase-deficient individuals. These results suggest that one of the primary consequences of telomere attrition and telomerase deficiencies could be the loss of NK immune function. This notion is compatible with the reported age-related decline in the number and function of these cells [43]. Of note, in some individuals the estimated telomere length in mature NK/T cells was near the predicted minimal telomere length (represented as a shaded area in Figure 1 and Figure 2) meaning that on average each chromosome end in those cells has fewer than 1 kb of telomere repeats.

Despite the finding that at birth telomeres in lymphocytes are longer than in granulocytes and despite the selective expression of telomerase in cells of the lymphoid lineage upon activation [22], [44], T lymphocytes displayed a sharp decline in telomere length with age. The steady decline in the telomere length in T cells likely contributes to compromised adaptive immunity in the elderly [45] and in individuals with telomerase deficiencies [46]. Interestingly, the narrowest telomere length distribution in leukocyte subsets from healthy individuals was observed in the memory T cell compartment, pointing to a possible role of telomere length in shaping the T cell repertoire and immune memory.

Our study of gender specific differences in telomere length confirmed previous observations that telomere lengths on average appear to be somewhat longer in females than in males [47]. The fact that this trend is already seen at birth raises questions that warrant further investigation: do females have fewer HSC at birth? Do female HSC have a higher replicative potential because of longer telomeres? Do stem cells in females have higher telomerase activity, possibly influenced by levels of sex hormones [48] or do other factors explain the longer telomeres in female leukocytes?

Epidemiological studies have been conducted to examine the potential validity of using relative leukocyte telomere length as a disease or aging associated biomarker. Interest in this area has greatly increased following recent reports of associations of shorter leukocyte telomere lengths with morbidity (such as cardiovascular disease or diabetes reviewed in [49]) and in response to external factors such as chronic stress [50]. More data is needed to confirm these findings and establish whether shorter leukocyte telomere lengths are associated with overall increased mortality in older adults [51], [52] and whether the increased risk of infection such as pneumonia in elderly individuals differs significantly in relation to their telomere lengths.

In conclusion, the data presented here contribute valuable base-line information regarding the telomere length in subpopulations of leukocytes during normal human ageing. This information will be a useful reference in studies of a variety of health conditions. Our data show that suppression of half of telomerase levels over a lifetime can severely compromise the telomere homeostasis of granulocytes as a surrogate marker for HSCs, and of immune cells. This likely is a dominant factor in the serious impairment of cell function and proliferative capacity that has been documented in telomerase deficient individuals. It seems possible that more effective short-term inhibition of telomerase could compromise the function of hematopoietic cells more acutely. Most likely, limitations imposed by progressive telomere loss act as a tumor suppressor mechanism in long-lived animals [42]. If so, caution is also needed for strategies that aim to rejuvenate older cells by reactivation of telomerase. The telomere length data described in this paper provide reference data for therapeutic strategies that target telomerase and for further studies on the role of telomeres and telomerase in normal aging and a variety of pathological conditions.

## Materials and Methods

### Ethical statement

All subjects enrolled in this study in Vancouver signed informed consent forms that were approved by the University of British Columbia (BC) and BC Cancer Agency Research Ethics Board. All samples from patients outside Vancouver were obtained with informed consent and approval of local ethical review boards in accordance with the Declaration of Helsinki.

### Healthy donors, telomerase-deficient individuals, and non-carrier relatives

Anonymous cord blood samples were obtained from healthy full term births with parental informed consent. Since no associate information was available for these samples, gender testing was performed by FISH as described below.

Anonymous peripheral blood samples were obtained from 835 healthy individuals between the ages of 6 months to 102 years of age screened for clotting disorders; samples where no clotting disorders were found were made available for study; only gender and age information were provided.

Samples from 60 individuals with confirmed telomerase deficiencies due to heterozygous mutations for either the telomerase reverse transcriptase (*hTERT*) or the RNA template (*hTERC*) gene and their 37 (non-carrier) relatives were included in our analysis and were described previously, (mean ages for both groups were 41 and 45 years respectively [25], [53]–[59]; all 97 participants or their guardians provided written informed consent in accordance with the Declaration of Helsinki.

### Cord blood gender determination

X and Y chromosome specific FISH was preformed as previously described [60]. Briefly, nucleated cord blood cells were fixed with methanol–acetic acid then dropped onto slides. Slides were fixed with formaldehyde, treated with pepsin, and dehydrated with ethanol. The hybridization mix containing fluorescently labeled peptide nucleic acid (PNA) probes specific for centromere repeats of respectively the X chromosome and the long arm of the Y chromosome were added to the slides. Following denaturation of DNA at 80°C for 3 minutes slides were incubated at room temperature for 30 minutes, washed, counterstained with DAPI and mounted using DABCO anti-fading reagent (Sigma Aldrich). Images were acquired and analyzed as previously described [60].

### Mutational analysis

*hTERT* and *hTERC* genotyping was performed as described previously [25], [53]–[59].

### Telomere length measurements by flow FISH

Telomere length measurements using automated multicolor flow-fluorescence in situ hybridization (flow FISH) was performed as described [61]. Briefly, white blood cells (WBCs) were isolated by osmotic lysis of erythrocytes in whole blood using NH_4_Cl. The WBCs were then mixed with bovine thymocytes of known telomere length (which serve as an internal control), denatured in formamide at 87°C, and hybridized with a fluorescein-conjugated (CCCTAA)_3_ peptide nucleic acid (PNA) probe specific for telomere repeats and counterstained with LDS751 DNA dye. The fluorescence intensity in, granulocytes, total lymphocytes and lymphocyte subsets defined by labeled antibodies specific for CD20, CD45RA and CD57 relative to internal control cells and unstained controls was measured on a FACSCalibur instrument (Becton Dickinson) to calculate the median telomere length from duplicate measurements. Further details regarding telomere length measurements and data sets are described in Online Supplementary Material as well as depicted in Figure S1.

Some of the total 835 healthy subjects did not have sufficient cells for analyses of one or more of the cell subsets tested: granulocytes, B lymphocytes, or mature NK/T cells. Specific improvements were developed during the 8 year of the healthy donor study allowing for the testing of additional cell subsets (B and mature NK/T [62]) explaining why fewer measurements are reported for these subsets. In addition, a slight modification was made to the cell lysis protocol: from a semi-automated small volume lysis in a 96 well format [63]) to the current larger volume individual sample lysis [61]. The data obtained during these two experimental periods were first analyzed separately to test for differences between the first and second data sets. Briefly, both data sets were found to have comparable telomere length distributions over age, with a small but notable decrease in the calculated granulocyte telomere length together with a decrease in the range of granulocyte telomere length values in the second data set (Figure S1). These differences may be explained by the protocol improvements in the second set that resulted in a better resolution of signal and a reduction in the background fluorescence observed in cell types with large volumes of cytoplasm. Since the ranges lower limits were similar between the two data sets and since the majority of the data for both sets falls within the same 10^th^ to 90^th^ percentile range, the two sets were merged and analyzed together for curve fitting models and reference comparisons.

### Statistical analyses

Analyses were performed using Microsoft Excel (Microsoft Office 2007), GraphPad Prism (version 4) and R (version 2.6.1, 2007, The R Foundation for Statistical Computing); t-Tests were two-tailed and performed on data with a normal distribution (KS test). Linear modeling (lm function in the R language) was used to carry out the regression analysis and estimate the piecewise linear curves with breakpoints hinged at 1 and 18 years of age. We found that this gave the best fit with the least mean square error and more consistent error distribution across the age ranges as compared to using a number of polynomial fits (linear, quadratic, cubic or quartic). The 99^th^, 90^th^, 10^th^ and 1^st^ percentile curves were obtained by vertical shift of the estimated regression curve to span the desired number of data points in the primary data set of healthy subjects (n = 835) that were representative for this segment of distribution. To compare the telomere lengths of one population against another, the ANOVA function in the R language was employed to test if the data was from the same or 2 different model fits [64].

### Online supplementary material

Figure S1 depicts data from two consecutive experimental periods separately for lymphocytes and granulocytes respectively, and displays the previous quadratic curve fitting model (first ∼400 data points) compared to the current three piece-wise linear regression model (for which first and second data sets were combined) used in Figure 2, Figure 3, and Figure 4.

Figure S2 complements Figure 2 and highlights the telomere length skewing specifically seen in CD45RA+ CD20− lymphocytes of older individuals, where a higher proportion of terminally differentiated lymphocytes are likely present within the cell population with this phenotype. Further, Figure S2 depicts the skewing observed in telomerase heterozygous individuals, comparable to that seen in healthy older individuals (over 75 years of age).

Figure S3 complements Figure 3 and depicts gender segregated telomere length data measured by flow FISH for all leukocyte cell subsets tested, together with the model fit statistical test results from these analyses.

Figure S4 complements Figure 4 and depicts further analysis of leukocyte telomere length data from direct relatives, siblings or parents of telomerase heterozygous individuals. From this relatively small group, although a trend towards shorter MTLs is observed, no statistical difference between the groups and no statistically significant difference compared to healthy individuals was detected (Table S5).

Table S1 displays the complete telomere length data sets. Duplicate leukocyte telomere length data were collected over an eight year period and analyzed on two occasions (first analysis after 391 samples and the present analysis after next 445 samples). For the first set of data, freshly isolated nucleated blood cells were used whereas for the second set, nucleated cells were frozen prior to flow FISH (see Materials and Methods and data set comparisons, Figure S1). No marked differences in the calculated telomere length between the two data sets were observed for lymphocytes or lymphocyte subsets. Although the granulocyte telomere length values were slightly lower and more narrowly distributed in the second data set, the overall results were pooled for the current analysis.

Table S2 displays telomere length cell subset correlations at different age ranges. This table complements Figure 2 and Figure S2. It displays the correlative r values between paired cell population telomere length values. The cell population chosen as a reference has a set value of 1.

Tables S3, S4 and S5 display the complete statistical ANOVA analysis results for comparing telomere lengths of leukocyte subsets in cord blood (at birth), and comparing the two linear models (an estimate of the entire population) and an estimate of where factor “X” (gender for example) was taken into consideration and showed a significant difference.

## Supporting Information

Figure S1 Comparison between leukocyte telomere length data sets and between statistical models over the study period. A. In the first study period, median lymphocyte and granulocyte telomere length measurements determined by flow FISH in 391 healthy individuals from birth (umbilical cord blood) to 102 years of age (blue dots) with reference curves derived from a quadratic function which provided a better fit than linear regression models over the entire age-range. Quadratic model distribution curves represent the projected modeled percentiles of the data: 99th (red), 90th (green top), 50th (green centre), 10th (green bottom) and 1st (blue) percentile of telomere range distribution per age. B. Median lymphocyte and granulocyte telomere length measurements in 391 consecutive individuals after 96 well format red cell lysis (Data set 1, blue dots) and 444 other individuals (Data set 2, black dots) after large volume red cell lysis. C. In the combined data set, median lymphocyte and granulocyte telomere length measurements from the combined data sets with piece-wise linear model percentile estimates: 99th (red), 90th (dashed green top), 50th (green centre), 10th (dashed green bottom) and 1st (blue) percentile of the telomere range distribution at any given age. Three linear regression segments were connected at respectively one year and eighteen years of age, to give better representation of the data at younger and older age ranges; insets represent magnifications of the predicted telomere length distribution between birth and five years of age.
(EPS)

Figure S2 Comparison of median telomere length (MTL) in CD45RA+CD20− “Naïve” T lymphocytes and other nucleated blood cell types from the same individual over four distinct age groups. This table complements Figure 2 and Figure S2A. It displays the correlative r values between paired cell population telomere length values. Age-related changes in the relationship between the telomere length (in kb) in “naïve” T lymphocytes and the indicated cell types (granulocytes, memory T lymphocytes (CD45RA−CD20−) or B lymphocytes (CD45RA+CD20+). The cell population chosen as a reference has a set value of 1. Each point represents paired measurements for the two leukocyte subsets within the same healthy individual; individuals are coded within age group as follows: cord blood samples and below one year of age (n = 60, red), between one and eighteen years of age (n = 171, black), adults between nineteen and seventy five years of age (n = 431, green) and elderly, over seventy five years of age (n = 173, orange). Paired measurements are represented for: A. healthy individuals and B. telomerase heterozygous individuals.
(EPS)

Figure S3 Gender differences in telomere length between leukocyte subpopulations. Median telomere length values in leukocyte subpopulations from 835 healthy females and males ranging from newborns (umbilical cord blood) to hundred years of age determined by flow FISH. The following white blood cell subtypes were tested: lymphocytes, granulocytes, B lymphocytes (CD20+), “naïve” T lymphocytes (CD45RA−pos CD20+), memory T lymphocytes (CD45RA−) and mature NK/T cells (CD57+). Curves representing the 50th percentile curve of the modeled median telomere length per age for females (red) versus males (blue) are shown. Three linear segments connected at one year and eighteen years of age were used. ANOVA test P values for the difference between females and males for each gender specific subset were: lymphocytes P = 1.56×10−5, granulocytes P = 0.37, B lymphocytes P = 0.0018, “naïve” T lymphocytes P = 3.7×10−7, memory T lymphocytes P = 0.0012, mature NK/T cells P = 0.0028 respectively. (for complete analysis details, see Table S5).
(EPS)

Figure S4 Leukocyte telomere lengths of non-carrier siblings or parents of telomerase deficient individuals. The telomere length distribution in healthy individuals (Figure 2) was used to plot the median telomere length values obtained with cells from: leukocytes from 8 non-carrier siblings (cyan), 7 non-carrier parents (orange) of telomerase-deficient patients and 21 direct relatives of idiopathic pulmonary fibrosis telomerase-deficient patients (sibling or parent status unknown).
(EPS)

Table S1 Complete telomere length data sets from healthy individuals. Leukocyte median telomere length data (MTL, kb) were collected over an eight year period and analyzed on two occasions (first analysis after 391 samples and the present analysis after another 445 samples). For the first set of data, freshly isolated nucleated blood cells were used whereas for the second set, nucleated cells were frozen prior to flow FISH (see Online Supplementary Material on flow FISH method for details and Figure S1 for data set comparisons). No marked differences in the calculated telomere length between the two data sets were observed for lymphocytes or lymphocyte subsets (not shown). Although the granulocyte telomere length values were slightly lower and more narrowly distributed in the second data set, the overall results were pooled for the current analysis.
(XLS)

Table S2 Telomere length cell subset correlations at different age ranges. This table complements Figure 2 and Figure S2. It displays the correlative r values between paired cell population telomere length values. The cell population chosen as a reference has a set value of 1.
(DOC)

Table S3 ANOVA results summary table on the effect of gender on cord blood telomere length measurements. Effect of gender test of cord blood samples, in reference to Figure 3A; One way ANOVA with Tukey's multiple comparison test (Table S4).
(DOC)

Table S4 ANOVA post test results summary table on the effect of gender on cord blood telomere length measurements. One way ANOVA with Tukey's multiple comparison test results of the effect of gender on cord blood telomere length measurements.
(DOC)

Table S5 ANOVA results summary table on the effects of gender on telomere length measurements. This table summarizes the results comparing the two linear models (an estimate of the entire population) and an estimate of where factor “X” (gender for example) was taken into consideration and showed a significant difference. The following factors were tested: effect of gender (total population); effect of telomerase deficiency; effect for relatives of telomerase deficient individuals; effect of gene affected *TERT* or *TERC* for heterozygous individuals or relatives; effect of family relationship to heterozygous individual (sibling or parent). A linear model was applied to fit the telomere length variation with age to a piece-wise linear function with two age breakpoints (age 1and age 18). An ANOVA (F-test) of the model fit against a model that also takes into consideration the gender of the individual and was applied and showed that there was a significant difference in gender on telomere length (P = 3.7e-07). The ANOVA compares the two linear models (an estimate of the entire population (one linear fit) and an estimate where gender (or another factor) was taken into consideration (two linear fits to the data)) and showed a significance difference (i.e 2 fits are better than 1 fit, or differences in gender; which can be extended to say a difference in female gender or just as equally male gender.) With reference to the ANOVA tables, the first line shows the Residual degree of freedom (Res.Df) and Residual sum of squares (RSS) for the first model (no difference in gender). The second line (difference in gender model) shows in addition, the degree of freedom for the model, sum of squares, the F-value and the probability of the F-statistics (Pr (>F). If this value is less than 0.05, then there is a good chance that gender (for example) has an effect on telomere length.
(DOC)
