# Supplementary material for: Collapse of Telomere Homeostasis in Hematopoietic Cells Caused by Heterozygous Mutations in Telomerase Genes
Source: PLoS Genet. 2012 May 17;8(5):e1002696. doi: 10.1371/journal.pgen.1002696 (PMC3355073; doi:10.1371/journal.pgen.1002696)
Supplement: Table S2 — Telomere length cell subset correlations at different age ranges. This table complements Figure 2 and Figure S2. It displays the correlative r values between paired cell population telomere length values. The cell population chosen as a reference has a set value of 1. (DOC) [file pgen.1002696.s006.doc]

**Table S2. Telomere length cell subset correlations at different age ranges**

| **MTL correlation: grans vs (r values) ***  **age:** | **lymphocytes** | **granulocytes** | **CD20+** | **CD45RA+ CD20-** | **CD45RA-** |
| --- | --- | --- | --- | --- | --- |
| **<1** | 0.967 | 1 | 0.960 | 0.988 | 0.968 |
| **1-18** | 0.775 | 1 | 0.908 | 0.747 | 0.822 |
| **19-102** | 0.799 | 1 | 0.82 | 0.761 | 0.807 |
| **MTL correlation: “Naïve” vs (r values) ^#^**  **age:** | **lymphocytes** | **granulocytes** | **CD20+** | **CD45RA+ CD20-** | **CD45RA-** |
| **<1** | 0.976 | 0.988 | 0.959 | 1 | 0.976 |
| **1-18** | 0.975 | 0.747 | 0.908 | 1 | 0.822 |
| **19-75** | 0.940 | 0.724 | 0.778 | 1 | 0.840 |
| **>75** | 0.935 | 0.660 | 0.564 | 1 | 0.793 |

MTL median telomere length

* see Figure 2C

# see Figure S2

Note: CD57+ mature NK/T cell correlation not done due to insufficient data over all age distributions for accurate statistical estimate
