# Supplementary material for: Collapse of Telomere Homeostasis in Hematopoietic Cells Caused by Heterozygous Mutations in Telomerase Genes
Source: PLoS Genet. 2012 May 17;8(5):e1002696. doi: 10.1371/journal.pgen.1002696 (PMC3355073; doi:10.1371/journal.pgen.1002696)
Supplement: Table S3 — ANOVA results summary table on the effect of gender on cord blood telomere length measurements. Effect of gender test of cord blood samples, in reference to Figure 3A; One way ANOVA with Tukey's multiple comparison test (Table S4). (DOC) [file pgen.1002696.s007.doc]

**Table S3. ANOVA results summary table.**

| **One way ANOVA table Cord Blood (n=58)** | **SS** | **Df** | **MS** | **F** | **P value** |
| --- | --- | --- | --- | --- | --- |
| **Treatment (between leukocyte subsets)** | 30.52 | 4 | 7.630 |  |  |
| **Residual (within leukocyte subsets)** | 356.9 | 265 | 1.347 |  |  |
| **Total** | 387.4 | 269 |  |  |  |
| **Test Results** |  |  |  | 5.7 | 0.0002*** |

*** P<0.001
