# Supplementary material for: Collapse of Telomere Homeostasis in Hematopoietic Cells Caused by Heterozygous Mutations in Telomerase Genes
Source: PLoS Genet. 2012 May 17;8(5):e1002696. doi: 10.1371/journal.pgen.1002696 (PMC3355073; doi:10.1371/journal.pgen.1002696)
Supplement: Table S4 — ANOVA post test results summary table on the effect of gender on cord blood telomere length measurements. One way ANOVA with Tukey's multiple comparison test results of the effect of gender on cord blood telomere length measurements. (DOC) [file pgen.1002696.s008.doc]

**Table S4. ANOVA results summary table, post test.**

| **Tukey’s Multiple comparison post -test** | **Mean Diff.** | **q** | **95%CI of diff** | **P<0.05?** |
| --- | --- | --- | --- | --- |
| **Lymphocyte vs Granulocyte** | 0.8296 | 5.254 | 0.2115 to 1.448 | ** |
| **Granulocyte vs CD45RA-CD20- lymphocyte** | -0.9574 | 6.063 | -1.575 to -0.3393 | *** |
| **Granulocyte vs CD45RA+CD20- lymphocyte** | -0.6852 | 4.339 | -1.303 to -0.0671 | * |
| **Granulocyte vs CD45RA+CD20+ lymphocyte** | -0.4648 | 2.943 | -1.083 to 0.1533 | ns |

ns: not significant

* P<0.05
** P<0.01
*** P<0.001
