# Supplementary material for: Collapse of Telomere Homeostasis in Hematopoietic Cells Caused by Heterozygous Mutations in Telomerase Genes
Source: PLoS Genet. 2012 May 17;8(5):e1002696. doi: 10.1371/journal.pgen.1002696 (PMC3355073; doi:10.1371/journal.pgen.1002696)
Supplement: Table S5 — ANOVA results summary table on the effects of gender on telomere length measurements. This table summarizes the results comparing the two linear models (an estimate of the entire population) and an estimate of where factor “X” (gender for example) was taken into consideration and showed a significant difference. The following factors were tested: effect of gender (total population); effect of telomerase deficiency; effect for relatives of telomerase deficient individuals; effect of gene affected TERT or TERC for heterozygous individuals or relatives; effect of family relationship to heterozygous individual (sibling or parent). A linear model was applied to fit the telomere length variation with age to a piece-wise linear function with two age breakpoints (age 1and age 18). An ANOVA (F-test) of the model fit against a model that also takes into consideration the gender of the individual and was applied and showed that there was a significant difference in gender on telomere length (P = 3.7e-07). The ANOVA compares the two linear models (an estimate of the entire population (one linear fit) and an estimate where gender (or another factor) was taken into consideration (two linear fits to the data)) and showed a significance difference (i.e 2 fits are better than 1 fit, or differences in gender; which can be extended to say a difference in female gender or just as equally male gender.) With reference to the ANOVA tables, the first line shows the Residual degree of freedom (Res.Df) and Residual sum of squares (RSS) for the first model (no difference in gender). The second line (difference in gender model) shows in addition, the degree of freedom for the model, sum of squares, the F-value and the probability of the F-statistics (Pr (>F). If this value is less than 0.05, then there is a good chance that gender (for example) has an effect on telomere length. (DOC) [file pgen.1002696.s009.doc]

**Table S5. ANOVA results summary tables, group tests.**

| **Group** | **Cell subset Telomere Length** | **Model** | **Res.Df** | **RSS** | **Df** | **Sum of Squares** | **F** | **Pr (>F)** |
| --- | --- | --- | --- | --- | --- | --- | --- | --- |
| **Gender** | Lymphocytes | 1 | 831 | 778.6 |  |  |  |  |
|  |  | 2 | 827 | 753.1 | 4 | 25.5 | 6.9 | 1.56x10^-5^*** |
|  | Granulocytes | 1 | 805 | 964.4 |  |  |  |  |
|  |  | 2 | 801 | 959.3 | 4 | 5.08 | 1.1 | 0.37 |
|  | CD45RA+CD20- Lymphocytes | 1 | 829 | 984.9 |  |  |  |  |
|  |  | 2 | 825 | 943.5 | 4 | 41.4 | 9.0 | 3.7x10^-7^*** |
|  | CD45RA-CD20- Lymphocytes | 1 | 829 | 684.5 |  |  |  |  |
|  |  | 2 | 825 | 669.7 | 4 | 14.8 | 4.6 | 0.0012*** |
|  | CD45RA+CD20+ Lymphocytes | 1 | 740 | 680.1 |  |  |  |  |
|  |  | 2 | 736 | 665.3 | 4 | 15.7 | 4.3 | 0.0018*** |
|  | CD45RA+CD57+ Lymphocytes | 1 | 559 | 742.2 |  |  |  |  |
|  |  | 2 | 555 | 721 | 4 | 21.26 | 4.1 | 0.0028*** |
| **Tel +/- individuals (n=60)** | Lymphocytes | 1 | 891 | 1225.8 |  |  |  |  |
|  |  | 2 | 888 | 811.8 | 3 | 413.9 | 150.9 | <2.2x10^-16^*** |
|  | Granulocytes | 1 | 860 | 1413.6 |  |  |  |  |
|  |  | 2 | 857 | 993.8 | 3 | 419.4 | 120.6 | <2.2x10^-16^*** |
|  | CD45RA+CD20- Lymphocytes | 1 | 887 | 1564.1 |  |  |  |  |
|  |  | 2 | 884 | 1033.9 | 3 | 530.2 | 151.1 | <2.2x10^-16^*** |
|  | CD45RA-CD20- Lymphocytes | 1 | 887 | 989.1 |  |  |  |  |
|  |  | 2 | 884 | 706 | 3 | 283.1 | 118.2 | <2.2x10^-16^*** |
|  | CD45RA+CD20+ Lymphocytes | 1 | 798 | 1104.9 |  |  |  |  |
|  |  | 2 | 795 | 710.1 | 3 | 394 | 146.8 | <2.2x10^-16^*** |
|  | CD45RA+CD57+ Lymphocytes | 1 | 603 | 1001.9 |  |  |  |  |
|  |  | 2 | 600 | 759.9 | 3 | 242 | 63.7 | <2.2x10^-16^*** |
| **Tel +/+ relatives (n=37)** | Lymphocytes | 1 | 868 | 816.1 |  |  |  |  |
|  |  | 2 | 865 | 801.2 | 3 | 14.9 | 5.3 | 0.001175** |
|  | Granulocytes | 1 | 840 | 1010.6 |  |  |  |  |
|  |  | 2 | 837 | 982.1 | 3 | 28.4 | 8.1 | 2.63x10^-5^*** |
|  | CD45RA+CD20- Lymphocytes | 1 | 866 | 1038.2 |  |  |  |  |
|  |  | 2 | 863 | 1017.8 | 3 | 20.4 | 5.7 | 0.000671*** |
|  | CD45RA-CD20- Lymphocytes | 1 | 866 | 719.5 |  |  |  |  |
|  |  | 2 | 863 | 704.4 | 3 | 15.1 | 6.1 | 0.000385*** |
|  | CD45RA+CD20+ Lymphocytes | 1 | 778 | 720.2 |  |  |  |  |
|  |  | 2 | 775 | 704.3 | 3 | 15.9 | 5.8 | 0.0006188*** |
|  | CD45RA+CD57+ Lymphocytes | 1 | 587 | 780.6 |  |  |  |  |
|  |  | 2 | 584 | 767.3 | 3 | 13.3 | 3.4 | 0.01828*** |
| ***TERT* +/- individuals (n=37)** | Lymphocytes | 1 | 868 | 1044.4 |  |  |  |  |
|  |  | 2 | 865 | 797.4 | 3 | 247.0 | 89.3 | <2.2x10^-16^*** |
|  | Granulocytes | 1 | 837 | 1230.4 |  |  |  |  |
|  |  | 2 | 834 | 982.4 | 3 | 248 | 70.2 | <2.2x10^-16^*** |
|  | CD45RA+CD20- Lymphocytes | 1 | 865 | 1331.4 |  |  |  |  |
|  |  | 2 | 862 | 1012.9 | 3 | 318.5 | 90.3 | <2.2x10^-16^*** |
|  | CD45RA-CD20- Lymphocytes | 1 | 865 | 866.6 |  |  |  |  |
|  |  | 2 | 862 | 698.6 | 3 | 168.0 | 69.1 | <2.2x10^-16^*** |
|  | CD45RA+CD20+ Lymphocytes | 1 | 776 | 933.3 |  |  |  |  |
|  |  | 2 | 773 | 700.2 | 3 | 233.1 | 85.8 | <2.2x10^-16^*** |
|  | CD45RA+CD57+ Lymphocytes | 1 | 587 | 921.2 |  |  |  |  |
|  |  | 2 | 584 | 750.6 | 3 | 170.6 | 44.2 | <2.2x10^-16^*** |
| ***TERC* +/- individuals (n=23)** | Lymphocytes | 1 | 854 | 979.3 |  |  |  |  |
|  |  | 2 | 851 | 791.1 | 3 | 188.17 | 67.5 | <2.2x10^-16^*** |
|  | Granulocytes | 1 | 828 | 1167.5 |  |  |  |  |
|  |  | 2 | 825 | 974.6 | 3 | 192.8 | 54.4 | <2.2x10^-16^*** |
|  | CD45RA+CD20- Lymphocytes | 1 | 851 | 1241.5 |  |  |  |  |
|  |  | 2 | 848 | 1003.1 | 3 | 238.4 | 67.2 | <2.2x10^-16^*** |
|  | CD45RA-CD20- Lymphocytes | 1 | 851 | 819.4 |  |  |  |  |
|  |  | 2 | 848 | 690.8 | 3 | 128.7 | 52.6 | <2.2x10^-16^*** |
|  | CD45RA+CD20+ Lymphocytes | 1 | 762 | 872.2 |  |  |  |  |
|  |  | 2 | 759 | 690.7 | 3 | 181.4 | 66.5 | <2.2x10^-16^*** |
|  | CD45RA+CD57+ Lymphocytes | 1 | 575 | 833.4 |  |  |  |  |
|  |  | 2 | 572 | 749.70 | 3 | 83.7 | 21.3 | 4.4x10^-13^*** |
| ***TERT* +/+ relatives (n=11)** | Lymphocytes | 1 | 842 | 791.0 |  |  |  |  |
|  |  | 2 | 839 | 781.0 | 3 | 9.91 | 3.55 | 0.0142* |
|  | Granulocytes | 1 | 815 | 976.6 |  |  |  |  |
|  |  | 2 | 812 | 967.2 | 3 | 9.39 | 2.63 | 0.0492* |
|  | CD45RA+CD20- Lymphocytes | 1 | 840 | 1000.8 |  |  |  |  |
|  |  | 2 | 837 | 987.3 | 3 | 13.6 | 3.83 | 0.0009* |
|  | CD45RA-CD20- Lymphocytes | 1 | 840 | 695.7 |  |  |  |  |
|  |  | 2 | 837 | 686.6 | 3 | 9.05 | 3.7 | 0.0119* |
|  | CD45RA+CD20+ Lymphocytes | 1 | 751 | 692.2 |  |  |  |  |
|  |  | 2 | 748 | 683.5 | 3 | 8.7 | 3.16 | 0.0242* |
|  | CD45RA+CD57+ Lymphocytes | 1 | 569 | 749.3 |  |  |  |  |
|  |  | 2 | 566 | 744.1 | 3 | 5.19 | 1.32 | 0.2679 |
| ***TERC* +/+ relatives (n=6)** | Lymphocytes | 1 | 837 | 786.1 |  |  |  |  |
|  |  | 2 | 834 | 781.9 | 3 | 4.25 | 1.51 | 0.2100 |
|  | Granulocytes | 1 | 811 | 970.5 |  |  |  |  |
|  |  | 2 | 808 | 968.8 | 3 | 1.59 | 0.44 | 0.7236 |
|  | CD45RA+CD20- Lymphocytes | 1 | 835 | 994.2 |  |  |  |  |
|  |  | 2 | 832 | 986.9 | 3 | 7.25 | 2.04 | 0.107 |
|  | CD45RA-CD20- Lymphocytes | 1 | 835 | 691.2 |  |  |  |  |
|  |  | 2 | 832 | 687.86 | 3 | 3.36 | 1.36 | 0.2550 |
|  | CD45RA+CD20+ Lymphocytes | 1 | 746 | 685.5 |  |  |  |  |
|  |  | 2 | 743 | 682.9 | 3 | 2.67 | 0.97 | 0.4075 |
|  | CD45RA+CD57+ Lymphocytes | 1 | 565 | 749.2 |  |  |  |  |
|  |  | 2 | 562 | 745.36 | 3 | 3.88 | 0.97 | 0.4046 |
| **Tel +/+ siblings of +/- individuals (n=8)** | Lymphocytes | 1 | 839 | 784.1 |  |  |  |  |
|  |  | 2 | 836 | 779.8 | 3 | 4.29 | 1.53 | 0.2046 |
|  | Granulocytes | 1 | 813 | 970.1 |  |  |  |  |
|  |  | 2 | 810 | 965.5 | 3 | 4.61 | 1.29 | 0.2767 |
|  | CD45RA+CD20- Lymphocytes | 1 | 837 | 992.43 |  |  |  |  |
|  |  | 2 | 834 | 985.63 | 3 | 6.8 | 1.92 | 0.1252 |
|  | CD45RA-CD20- Lymphocytes | 1 | 837 | 690.5 |  |  |  |  |
|  |  | 2 | 834 | 685.9 | 3 | 4.66 | 1.89 | 0.1298 |
|  | CD45RA+CD20+ Lymphocytes | 1 | 748 | 687.23 |  |  |  |  |
|  |  | 2 | 745 | 682.1 | 3 | 5.13 | 1.9 | 0.1333 |
|  | CD45RA+CD57+ Lymphocytes | 1 | 566 | 746.0 |  |  |  |  |
|  |  | 2 | 563 | 743.6 | 3 | 2.42 | 0.6 | 0.6085 |
| **Tel +/+ parents of +/- individuals (n=7)** | Lymphocytes | 1 | 838 | 786.4 |  |  |  |  |
|  |  | 2 | 836 | 780.8 | 2 | 5.56 | 3.0 | 0.0515 |
|  | Granulocytes | 1 | 812 | 974.6 |  |  |  |  |
|  |  | 2 | 810 | 969.8 | 2 | 4.9 | 2.04 | 0.1301 |
|  | CD45RA+CD20- Lymphocytes | 1 | 836 | 995.3 |  |  |  |  |
|  |  | 2 | 834 | 987.0 | 2 | 8.33 | 3.52 | 0.0301* |
|  | CD45RA-CD20- Lymphocytes | 1 | 836 | 691.1 |  |  |  |  |
|  |  | 2 | 834 | 686.7 | 2 | 4.36 | 2.65 | 0.0715 |
|  | CD45RA+CD20+ Lymphocytes | 1 | 747 | 688.5 |  |  |  |  |
|  |  | 2 | 745 | 684.1 | 2 | 4.41 | 2.40 | 0.0914 |
|  | CD45RA+CD57+ Lymphocytes | 1 | 566 | 746.1 |  |  |  |  |
|  |  | 2 | 564 | 743.8 | 2 | 2.27 | 0.86 | 0.4236 |

* P<0.05
** P<0.01
*** P<0.001
